# The State-Dependent Channel with a Rate-Limited Cribbing Helper

**DOI:** 10.3390/e26070570

**Published:** 2024-06-30

**Authors:** Amos Lapidoth, Yossef Steinberg

**Affiliations:** 1 Signal and Information Processing Laboratory, ETH Zurich, 8092 Zurich, Switzerland; 2 Department of Electrical and Computer Engineering, Technion—Israel Institute of Technology, Haifa 3200003, Israel; ysteinbe@technion.ac.il

**Keywords:** backward decoding, block Markov, causal, cribbing, helper, rate limited, state dependent channel

## Abstract

The capacity of a memoryless state-dependent channel is derived for a setting in which the encoder is provided with rate-limited assistance from a cribbing helper that observes the state sequence causally and the past channel inputs strictly causally. Said cribbing may increase capacity but not to the level achievable by a message-cognizant helper.

## 1. Introduction

An encoder for a state-dependent channel is said to have causal state information if the channel input $X_{i}$ it produces at time *i* may depend, not only on the message *m* it wishes to transmit, but also on the present and past channel states $S_{i}$ and $S^{i-1}$ (where $S^{i-1}$ stands for the states $S_{1},\ldots ,S_{i-1}$). Its state information is noncausal if, in addition to depending on the message, its time *i* input may depend on all the channel states: past $S^{i-1}$, present $S_{i}$, and future $S_{i+1}^{n}$ (where *n* denotes the blocklength, and $S_{i+1}^{n}$ stands for $S_{i+1},\ldots ,S_{n}$).

The former case was studied by Shannon [1], who showed that capacity can be achieved by what-we-now-call Shannon strategies. The latter was studied by Gel’fand and Pinsker [2], who showed that the capacity, in this case, can be achieved using binning [3].

As of late, there has been renewed interest in the causal case, but when the state information must be quantized before it is provided to the encoder [4]. While still causal, the encoder is not provided now with the state sequence $\left\{S_{i}\right\}$ directly, but rather with some “assistance sequence” $\left\{T_{i}\right\}$ describing it. Its time *i* output $X_{i}$ is now determined by the message *m* and by the present and past assistances $T^{i}$. The assistance sequence is produced by a helper, which observes the state sequence causally and produces the time *i* assistance $T_{i}$ based on the present and past states $S^{i}$ subject to the additional constraint that $T_{i}$ take values in a given finite set T whose cardinality is presumably smaller than that of the state alphabet S. (If the cardinality of T is one, the problem reduces to the case of no assistance; if it exceeds or equals the cardinality of S, the problem reduces to Shannon’s original problem because, in this case, $T_{i}$ can describe $S_{i}$ unambiguously.) We refer to the base-2 logarithm of the cardinality of T as the “help rate” and denote it $R_{h}$:

(1) $$\begin{array}{ccc} R_{h} & = & \log_{2}\left|T\right|. \end{array}$$

Three observations in [4] inspired the present paper:

1. Symbol-by-symbol quantizers are suboptimal: restricting $T_{i}$ to be a function of $S_{i}$ may reduce capacity.
2. Allowing $T_{i}$ to depend not only on $S^{i}$ but also on the message *m* may increase capacity.
3. If $T_{i}$ is allowed to depend on $S^{i}$, as well as on the transmitted message, then message-cognizant symbol-by-symbol helpers achieve capacity: there is no loss in capacity in restricting $T_{i}$ to be a function of $\left(m,S_{i}\right)$.

Sandwiched between the message-oblivious helper and the message-cognizant helper is the cribbing helper whose time-*i* assistance $T_{i}$ depends on $S^{i}$ and on the past symbols produced by the encoder

(2) $$\begin{array}{ccc} T_{i} & = & T_{i}\left(S^{i},X^{i-1}\right). \end{array}$$

**Figure 1:**
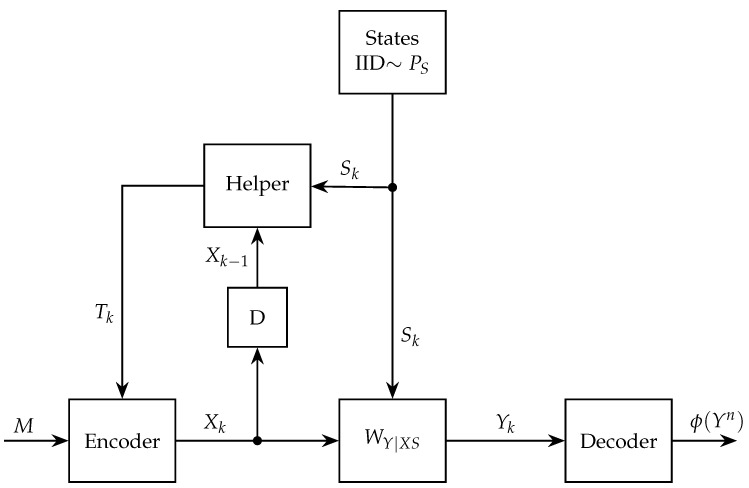
Communication over a state-dependent channel with a rate-limited causal cribbing helper.

Such a helper is depicted in Figure 1.

Since one can reproduce the channel inputs from the states and message, the cribbing helper cannot outperform the message-cognizant helper. And since the helper can ignore the past channel inputs, the cribbing capacity must be at least as high as that of the message-oblivious helper.

Here, we shall characterize the capacity with a cribbing helper and show that the above inequalities can be strict: the message-cognizant helper may outperform the cribbing helper, and the latter may outperform the message-oblivious helper (presumably because, thanks to the cribbing, it can learn something about the message). We further show that the capacity of the cribbing helper can be achieved using a block Markov coding scheme with backward decoding.

It is important to note that allowing the helper to crib does not render it a relay [5] because the helper does not communicate with the receiver. Therefore, our results do not have any bearing on the Relay problem.

It is also noteworthy that message-cognizant helpers are also advantageous in the noncausal case. For such helpers, capacity was recently computed in [6,7]. Cribbing, however, is somewhat less natural in this setting.

## 2. Problem Statement and Main Result

We are given a state-dependent discrete memoryless channel $W_{Y|XS}$ of finite input, output, and state alphabets X, Y, and S. When its input is x∈X and its state is s∈S, the probability of its output being y∈Y is $W_{Y|XS}\left(y|x,s\right)$. The states $\left\{S_{i}\right\}$ are drawn IID $∼P_{S}$, where $P_{S}$ is some given probability mass function (PMF) on the state alphabet S. Also given is some finite set T that we call the description alphabet. We shall assume throughout that its cardinality is at least 2

(3) |T|≥2

because otherwise the helper cannot provide any assistance.

Given some blocklength *n*, a rate-*R* message set is a set M whose cardinality is $2^{nR}$ (where we ignore the fact that the latter need not be an integer). A blocklength-*n* encoder for our channel comprises *n* mappings

(4) $$\begin{array}{c} f_{i}:M\times T^{i}\to X,\;i=1,\ldots ,n \end{array}$$

with the understanding that if the message to be transmitted is m∈M, and if the assistance sequence produced by the helper is $t^{n}\in T^{n}$, then the time *i* channel input produced by the encoder is

(5) $$\begin{array}{ccc} x_{i} & = & f_{i}\left(m,t^{i}\right) \end{array}$$

which we also denote $x_{i}\left(m,t^{i}\right)$. Here, $T^{i}$ denotes the *i*-fold Cartesian product

(6) $$\begin{array}{c} T^{i}=\underset{i\;\mathrm{times}}{\underset{︸}{T\times T\times \cdots \times T}} \end{array}$$

and $t^{j}$ denotes $t_{1},\ldots ,t_{j}$. A blocklength-*n* cribbing helper comprises *n* mapping

(7) $$\begin{array}{c} h_{i}:X^{i-1}\times S^{i}\to T,\;i=1,\ldots ,n \end{array}$$

with the understanding that—after observing the channel inputs $x_{1},\ldots ,x_{i-1}$ and the states $s_{1},\ldots ,s_{i}$—the helper produces the time *i* assistance

(8) $$\begin{array}{ccc} t_{i} & = & h_{i}\left(x^{i-1},s^{i}\right) \end{array}$$

which we also denote $t_{i}\left(x^{i-1},s^{i}\right)$.

Communication proceeds as follows: the helper produces the time-1 assistance $t_{1}$ that is given by $h_{1}\left(s_{1}\right)$, and the encoder then produces the first channel input $x_{1}=f_{1}\left(m,t_{1}\right)$. The helper then produces the time-2 assistance $t_{2}$ that is given by $h_{2}\left(x_{1},s^{2}\right)$, and the encoder then produces the second channel input $x_{2}=f_{2}\left(m,t^{2}\right)$, and so on.

The decoder is cognizant neither of the state sequence $s^{n}$ nor of the assistance sequence $t^{n}$: it is thus a mapping of the form

(9) $$\phi :Y^{n}\to M$$

with the understanding that, upon observing the output sequence $Y^{n}$, the decoder guesses that the transmitted message is $\phi (Y^{n})$.

Let $P_{e}=\mathrm{Pr}\left(\phi (Y^{n})\neq M\right)$ denote the probability of a decoding error when the message *M* is drawn uniformly from M. If $P_{e}<\epsilon$, then we say that the coding scheme is of parameters $\left(n,2^{nR},|T|,\epsilon \right)$ or that it is a $\left(n,2^{nR},|T|,\epsilon \right)$-scheme. A rate *R* is said to be achievable if, for every ϵ>0, there exist, for all sufficiently large *n*, schemes as above with $P_{e}<\epsilon$. The capacity of the channel is defined as the supremum of all achievable rates *R* and is denoted *C*.

Define

(10) $$C^{\left(I\right)}=\max\min\left\{I(UV;Y),I(U;X|VT)\right\}$$

where the maximum is over all finite sets U and V and over all joint distributions of the form

(11) $$P_{S}\;P_{UV}\;P_{T|VS}\;P_{X|UVT}\;W_{Y|XS}$$

with *T* taking values in the assistance alphabet T. (When writing Markov conditions and information theoretic quantities such as entropy and mutual information, we do not separate the variables with commas. We thus write H(XY), and not H(X,Y), for the joint entropy of *X* and *Y*. We do, however, introduce commas when this convention can lead to ambiguities; see, for example, (62).)

Our main result is stated next.

**Theorem** **1.** *The capacity C of the memoryless state-dependent channel with rate-limited cribbing helper equals $C^{\left(I\right)}$:*

(12) $$C=C^{\left(I\right)}.$$

*Moreover, the maximum in (10) can be achieved when:*

*1.*  *$P_{T|VS}$ and $P_{X|UVT}$ are both zero-one laws.*
*2.*  *The alphabet sizes of U and V are restricted to*
$$\begin{array}{ccc} \left|V\right| & \leq & L^{2}\;\left|S\right|\;\left(|T|-1\right)+L\\ \left|U\right| & \leq & L^{3}\;\left|T\right|\left(|X|-1\right)+L \end{array}$$
  *where L=|X||T||S|+1.*
*3.*  *The chain*
(13) V⊸– U⊸– (XTS)⊸– Y
  *is a Markov chain.*

(Henceforth, we use A⊸– B⊸– C to indicate that *A* and *C* are conditionally independent given *B* and, more generally, A⊸– B⊸– C⊸– D to indicate that A,B,C,D forms a Markov chain.)

The proof is given in Section 4.

**Remark** **1.** *The assumption of (3) notwithstanding, the theorem also holds in case |T|=1, which corresponds to no help.*

**Proof of Remark 1.**  When *T* is deterministic, $P_{X|UVT}$ equals $P_{X|UV}$, and the data processing inequality implies that I(UV;Y)≤I(X;Y), thus establishing that, in this case, $C^{\left(I\right)}$ is upper bounded by the capacity without state information, i.e.,

(14) $$\begin{array}{ccc} C^{\left(I\right)} & \leq & \max_{P_{X}}I\left(X;Y\right). \end{array}$$

Equality can be established by choosing *V* as null and *U* as *X*, a choice that results in I(UV;Y) being I(X;Y) and in I(U;X|VT) being H(X). □

**Remark** **2.** *As is to be expected, when |T|≥|S|, i.e., when T can describe S precisely, $C^{\left(I\right)}$ reduces to the Shannon strategies capacity $C^{\left(Sh\right)}$ of the channel with perfect causal state information at the transmitter:*

(15) $$C^{\left(\mathrm{Sh}\right)}=\max I\left(U;Y\right)$$

*where the maximization is over all the joint PMFs of the form $P_{S}\;P_{U}\;P_{X|US}\;W_{Y|XS}$ (and where, without altering the result of the maximization, we can restrict $P_{X|US}$ to be zero–one valued).*

**Proof of Remark 2.**  We first establish that $C^{\left(I\right)}$ is greater-equal $C^{\left(\mathrm{Sh}\right)}$. To that end, we set *T* to equal *S* and *V* to be null and then argue that, with this choice, the minimum of the two terms in (10) is the first, i.e., I(UV;Y) (which, because *V* is null, equals I(U;Y)). To that end, we calculate

(16)I(U;X|VT)=I(U;X|VT)(17)                   =I(U;X|S)(18)                   =I(U;XS)(19)                   =I(U;Y)

where the first equality holds because *V* is null, the second because *T* equals *S*, the third because *U* is independent of *S*, and the final inequality follows from the Data Processing inequality.

It remains to prove that

(20) $$\begin{array}{ccc} C^{\left(I\right)} & \leq & C^{\left(\mathrm{Sh}\right)} \end{array}$$

which always holds. To simplify our analysis, we assume the Markov condition (13), and we then upper-bound I(UV;Y) (which is an upper bound on the minimum in the definition (10) of $C^{\left(I\right)}$). Under this Markov condition, the maximum of I(UV;Y) can be achieved with *V* null, which we proceed to assume. The joint PMF of the remaining variables is then of the form

(21) $$P_{S}\;P_{U}\;P_{T|S}\;P_{X|UT}\;W_{Y|XS}.$$

We will show that—for every fixed $P_{U}$—to any choice of $P_{T|S}$ and $P_{X|UT}$ there corresponds a choice of $P_{X|US}$, which is feasible for the maximization defining $C^{\left(\mathrm{Sh}\right)}$ in (15) and that thus proves that $C^{\left(\mathrm{Sh}\right)}\geq I\left(U;Y\right)$.

To this end, we begin by expressing the channel from *U* to *Y* using (21) as

(22)PY|U=∑sPSPX|USWY|XS(23)        =∑sPS∑tPT|USPX|USTWY|XS(24)        =∑sPS∑tPT|SPX|UTWY|XS.

We then note that, for a fixed $P_{U}$, the mutual information I(U;Y) is thus determined by the |S|·|U| conditional PMFs of *X* given (S,U)=(s,u)

$${\left\{\sum_{t}P_{T|US}\left(t|u,s\right)\;P_{X|UT}\left(x|u,t\right)\right\}}_{(s,u)\in S\times U}.$$

These conditional PMFs are feasible for the maximization defining $C^{\left(\mathrm{Sh}\right)}$ in (15), thus demonstrating that $C^{\left(\mathrm{Sh}\right)}\geq I\left(U;Y\right)$. □

## 3. Example

We next present an example where the message-cognizant helper outperforms the cribbing helper and the latter outperforms the plain-vanilla causal helper. It is trivial to find cases where the three perform identically, e.g., when the state does not affect the channel. The example is borrowed from ([4], Example 7) (from which we also lift the notation).

The channel inputs, states, and outputs are binary tuples

(25) X=S=Y={0,1}×{0,1}

and are denoted (A,B), $\left(S^{\left(0\right)},S^{\left(1\right)}\right)$, and $\left(Y^{\left(0\right)},Y^{\left(1\right)}\right)$ respectively. The two components of the state are IID, each taking on the values 0 and 1 equiprobably. Given the state and input, the channel output is deterministically given by

(26) $$Y=\left(A,B⊕S^{\left(A\right)}\right).$$

The assistance is one-bit assistance, so T={0,1}.

As shown in ([4], Claim 8), the capacity with a message-cognizant helper is 2 bits, and with a message-oblivious helper log3. Here, we show that the capacity with a cribbing helper is strictly smaller than 2 bits and strictly larger than log3. All logarithms in this section are base-2 logarithms, and all rates are in bits.

We begin by showing the former. Recall that if *R* is achievable, then it must satisfy the constraints

(27) $$\begin{array}{ccc} R & \leq & I(UV;Y) \end{array}$$

(28) $$\begin{array}{ccc} R & \leq & I(U;X|VT). \end{array}$$

Recall also the form of the joint PMF

(29) $$P_{S}\;P_{V}\;P_{T|VS}\;P_{U|V}P_{X|UVT}\;W_{Y|XS}$$

and that we may assume that $P_{X|UVT}\left(x|u,v,t\right)$ is zero-one valued. Note that (29) implies

(30) ST⊸– V⊸– U

and consequently

(31) S⊸– TV⊸– U.

We will show that the above constraints cannot be both satisfied if R=2. To that end, we assume that

(32) $$\begin{array}{c} I(U;X|VT)=2 \end{array}$$

(it cannot be larger because $\left|X\right|=4$) and prove that

(33) $$\begin{array}{c} I(UV;Y)<2. \end{array}$$

Since Y is of cardinality 4, it suffices to show that

(34) H(Y|UV)>0.

In fact, it suffices to show that

(35) H(Y|UVT)>0

i.e., that there exist $u^{★},v^{★},t^{★}$ of positive probability for which

(36) $$H(Y|U=u^{★},V=v^{★},T=t^{★})>0.$$

This is what we proceed to do. We first show the existence of $v^{★}$ and $t^{★}$ for which $H(S|V=v^{★},T=t^{★})\geq 1$. Once this is established, we proceed to pick $u^{★}$.

Since $\left|X\right|=4$, (32) implies that

(37) $$P_{X|V=v,T=t}\;\mathrm{is}\;\mathrm{uniform}\;\forall \left(v,t\right).$$

Fix any $v^{★}$ (of positive probability). As we next argue, there must exist some $t^{★}$ for which $P_{S|V=v^{★},T=t^{★}}$ is not zero–one valued. Indeed, by (29), V⊥⊥S, so $H\left(S|V=v^{★}\right)=H\left(S\right)=2$ and

(38)H(S|T,V=v★)=H(S|V=v★)−I(S;T|V=v★)(39)                           =H(S)−I(S;T|V=v★)(40)                           ≥H(S)−H(T|V=v★)(41)                           ≥2−logT(42)                           =1

so there must exist some $t^{★}$ for which

(43) $$H(S|V=v^{★},T=t^{★})\geq 1.$$

We next choose $u^{★}$ as follows. Conditional on $V=v^{★},T=t^{★}$, the chance variable *U* has some PMF $P_{U|V=v^{★},T=t^{★}}$ (equal to $P_{U|V=v^{★}}$ by (29)) under which $X(U,v^{★},t^{★})$ is uniform; see (37). It follows that there exist $u_{0}$ and $u_{1}$ (both of positive conditional probability) such that

(44) $$\begin{array}{ccc} A(u_{0},v^{★},t^{★}) & = & 0 \end{array}$$

(45) $$\begin{array}{ccc} A(u_{1},v^{★},t^{★}) & = & 1 \end{array}$$

where we introduced the notation

(46) $$X\left(u,v^{★},t^{★}\right)=\left(A\left(u,v^{★},t^{★}\right),B\left(u,v^{★},t^{★}\right)\right).$$

Returning to (43), we note that it implies that

(47) $$\begin{array}{c} H\left(S^{\left(0\right)}|V=v^{★},T=t^{★}\right)>0\;\mathrm{or}\;H\left(S^{\left(1\right)}|V=v^{★},T=t^{★}\right)>0. \end{array}$$

In the former case, $H(Y|U=u_{0},V=v^{★},T=t^{★})$ is positive, and in the latter, $H(Y|U=u_{1},V=v^{★},T=t^{★})$ is positive. This establishes the existence of a triple $\left(u^{★},v^{★},t^{★}\right)$ for which (36) holds, and thus concludes the proof that the capacity with a cribbing encoder is smaller than 2. We next show that it exceeds log3.

To that end, we consider choosing

(48) $$U=(A,\tilde{U})$$

to be uniform over {0,1}×{0,1}, and we let σ be a Bernoulli–α random variable that is independent of *U* and of the channel, for some α∈[0,1] to be specified later. We further define

(49) $$\begin{array}{c} \tilde{V}=\left\{\begin{array}{ccc} A & \mathrm{if} & \sigma =1\\ 0\;\left(\mathrm{null}\right) & \mathrm{if} & \sigma =0 \end{array}\right. \end{array}$$

and

(50) $$V=(\tilde{V},\sigma ).$$

We choose the helper function h(s,v)—which can also be written as $h\left(\left(s^{\left(0\right)},s^{\left(1\right)}\right),\left(\tilde{v},\sigma \right)\right)$—to equal $s^{\left(\tilde{v}\right)}$, so

(51) $$T=S^{\left(\tilde{V}\right)}$$

and

(52) $$T=\left\{\begin{array}{cc} S^{\left(A\right)} & w.p.\alpha \\ S^{\left(0\right)} & w.p.1-\alpha . \end{array}\right.$$

Our encoder function f(u,v,t) ignores *v* and results in

(53) $$X^{\left(0\right)}=A,\;X^{\left(1\right)}=\tilde{U}⊕T$$

where $X=(X^{\left(0\right)},X^{\left(1\right)})$. That is,

(54) $$f\left((A,\tilde{U}),T\right)=\left(A,\tilde{U}⊕T\right).$$

Note that with the variables defined in (49)–(53), the Markov relations in item 3 of Theorem 1 hold.

We now proceed to calculate the rate bounds. For the RHS of (27), we have

(55) $$\begin{array}{ccc} I(UV;Y) & = & I(U\tilde{V}\sigma ;Y)\\ & \geq & I(U\tilde{V};Y|\sigma )\\ & = & \alpha \;I\left(U\tilde{V};Y|\sigma =1\right)+\left(1-\alpha \right)\;I\left(U\tilde{V};Y|\sigma =0\right)\\ & = & \alpha \;I\left(A\tilde{U};Y|\sigma =1\right)+\left(1-\alpha \right)\;I\left(A\tilde{U};Y|\sigma =0\right) \end{array}$$

where the last equality holds because if σ=0, then $\tilde{V}$ is null.

We next evaluate each of the terms on the RHS of (55) separately. When σ=1,

$$T=S^{\left(A\right)}$$

$$T=S^{\left(A\right)}X^{\left(1\right)}=\tilde{U}⊕S^{\left(A\right)}$$

(56) $$Y^{\left(1\right)}=X^{\left(1\right)}⊕S^{\left(A\right)}=\tilde{U}⊕S^{\left(A\right)}⊕S^{\left(A\right)}=\tilde{U}$$

so

(57) $$Y=\left(Y^{\left(0\right)},Y^{\left(1\right)}\right)=\left(A,\tilde{U}\right)$$

and

(58) $$\begin{array}{ccc} I(A\tilde{U};Y|\sigma =1) & = & H(U|\sigma =1)\\ & = & H(U)\\ & = & 2 \end{array}$$

where the second equality holds because σ is independent of *U*.

When σ=0,

$$\begin{array}{ccc} T & = & S^{\left(0\right)} \end{array}$$

$$\begin{array}{ccc} X & = & \left(A,\tilde{U}⊕S^{\left(0\right)}\right) \end{array}$$

(59) $$\begin{array}{ccc} Y & = & \left(A,\tilde{U}⊕S^{\left(0\right)}⊕S^{\left(1\right)}\right) \end{array}$$

so

(60) $$\begin{array}{ccc} I(A\tilde{U};Y|\sigma =0) & = & I(A\tilde{U};Y^{\left(0\right)}Y^{\left(1\right)}|\sigma =0)\\ & = & I(A\tilde{U};A,\tilde{U}⊕S^{\left(0\right)}⊕S^{\left(A\right)})\\ & = & I\left(A\tilde{U};A\right)+I\left(A\tilde{U};\tilde{U}⊕S^{\left(0\right)}⊕S^{\left(A\right)}|A\right)\\ & = & H\left(A\right)+\frac{1}{2}\;I\left(\tilde{U};\tilde{U}⊕S^{\left(0\right)}⊕S^{\left(0\right)}|A=0\right)+\frac{1}{2}\;I\left(\tilde{U};\tilde{U}⊕S^{\left(0\right)}⊕S^{\left(1\right)}|A=1\right)\\ & = & H\left(A\right)+\frac{1}{2}H\left(\tilde{U}\right)+0=\frac{3}{2}. \end{array}$$

From (58), (60), and (55), we obtain that the RHS of (27) satisfies

(61) $$I\left(UV;Y\right)\geq 2\alpha +\left(1-\alpha \right)\frac{3}{2}=\left(\alpha +3\right)/2.$$

Next, we evaluate the RHS of (28):

(62) $$\begin{array}{ccc} I(U;X|VT) & = & I(U;X|\tilde{V},\sigma ,T)\\ & = & \alpha \;I\left(U;X|\tilde{V},\sigma =1,T\right)+\left(1-\alpha \right)\;I\left(U;X|\tilde{V},\sigma =0,T\right)\\ & = & \alpha \;I\left(A\tilde{U};X|A,S^{\left(A\right)},\sigma =1\right)+\left(1-\alpha \right)\;I\left(A\tilde{U};A,\tilde{U}⊕S^{\left(0\right)}|S^{\left(0\right)},\sigma =0\right)\\ & = & \alpha \;I\left(\tilde{U};A,\tilde{U}⊕T|AS^{\left(A\right)},\sigma =1\right)+\left(1-\alpha \right)\;I\left(A\tilde{U};A,\tilde{U}⊕S^{\left(0\right)}|S^{\left(0\right)},\sigma =0\right)\\ & = & \alpha \;I\left(\tilde{U};\tilde{U}⊕T|AS^{\left(A\right)},\sigma =1\right)+\left(1-\alpha \right)\;H\left(A,\tilde{U}\right)\\ & = & \alpha \;H\left(\tilde{U}\right)+\left(1-\alpha \right)\;H\left(A,\tilde{U}\right)\\ & = & \alpha +(1-\alpha )\;2\\ & = & 2-\alpha . \end{array}$$

In view of (61) and (62), any rate *R* satisfying

(63) R≤min{(α+3)/2,2−α}

is achievable. Choosing α=1/3 (which maximizes the RHS of (63)), demonstrates the achievability of

(64) R=5/3

which exceeds log3.

## 4. Proof of Theorem 1

### 4.1. Direct Part

Pick a distribution as in (11), where $P_{T|SV}$ and $P_{X|UVT}$ are 0–1 laws, so

(65) $$\begin{array}{ccc} x & = & f(u,v,t) \end{array}$$

(66) $$\begin{array}{ccc} t & = & h(s,v) \end{array}$$

for some deterministic functions *f* and *h*. Extend these functions to act on *n*-tuples componentwise so that if s,v are *n*-tuples in $S^{n}$ and $V^{n}$, then t=h(s,v) indicates that *t* is an *n*-tuple in $T^{n}$ whose *i*-th component $t_{i}$ equals $h(s_{i},v_{i})$, where $s_{i}$ and $v_{i}$ are the corresponding components of *s* and *v*. Likewise, we write x=f(u,v,t).

To prove achievability, we propose a block Markov coding scheme with the receiver performing backward decoding. Although only the receiver is required to decode the message, in our scheme, the helper does too (but not with backward decoding, which would violate causality).

The transmission comprises *Bn*-length sub-blocks, for a total of Bn channel uses. The transmitted message *m* is represented by B−1 sub-messages $m_{1},\ldots ,m_{B-1}$, with each of the sub-messages taking values in the set $M\overset{\Delta }{=}\left\{1,2,\ldots ,2^{nR}\right\}$. The overall transmission rate is thus R(B−1)/B, which can be made arbitrarily close to *R* by choosing *B* very large. The B−1 sub-messages are transmitted in the first B−1 sub-blocks, with $m_{b}$ transmitted in sub-block *b* (for b∈[1:B−1]). Hereafter, we use $s^{\left(b\right)}$ to denote the state *n*-tuple affecting the channel in sub-block *b* and use $s_{i}^{\left(b\right)}$ to denote its *i*-component (with i∈[1:n]). Similar notation holds for $x^{\left(b\right)}$, $y^{\left(b\right)}$, etc.

We begin with an overview of the scheme, where we focus on the transmission in sub-blocks 2 through B−1: the first and last sub-blocks must account for some edge effects that we shall discuss later. Let *b* be in this range. The coding we use in sub-block *b* is superposition coding with the cloud center determined by $m_{b-1}$ and the satellite by $m_{b}$.

Unlike the receiver, the helper, which must be causal, cannot employ backward decoding: it decodes each sub-message at the end of the sub-block in which it is transmitted. Consequently, when sub-block *b* begins, it already has a reliable guess ${\hat{m}}_{b-1}$ of $m_{b-1}$ (based on the previous channel inputs $x^{\left(b-1\right)}$ it cribbed). The encoder, of course, knows $m_{b-1}$, so the two can agree on the cloud center $v^{\left(b\right)}\left(m_{b-1}\right)$ indexed by $m_{b-1}$. (We ignore for now the fact that ${\hat{m}}_{b-1}$ may, with small probability, differ from $m_{b-1}$.) The satellite is computed by the encoder as $u^{\left(b\right)}\left(m_{b}|m_{b-1}\right)$; it is unknown to the helper. The helper produces the sub-block *b* assistance $t^{\left(b\right)}$ based on the state sequence and the cloud center

(67) $$t^{\left(b\right)}=h\left(s^{\left(b\right)},v^{\left(b\right)}\left(m_{b-1}\right)\right).$$

(Since h(·,·) acts componentwise, this help is causal with the *i*-th component of $t^{\left(b\right)}$ being a function of the corresponding component $s_{i}^{\left(b\right)}$ of the state sequence and $v^{\left(b\right)}\left(m_{b-1}\right)$; it does not require knowledge of future states.)

For its part, the encoder produces the *n*-tuple

(68) $$x^{\left(b\right)}=f\left(u^{\left(b\right)}\left(m_{b}|m_{b-1}\right),v^{\left(b\right)}\left(m_{b-1}\right),t^{\left(b\right)}\right)$$

with causality preserved because $u^{\left(b\right)}\left(m_{b}|m_{b-1}\right)$ and $v^{\left(b\right)}\left(m_{b-1}\right)$ can be computed from $m_{b-1}$ and $m_{b}$ ahead of time, and because ***t*** is presented to the encoder causally and f(·) operates componentwise.

As to the first and last sub-blocks: In the first, we set $m_{0}$ as constant (e.g., $m_{0}=1$), so we have only one cloud center. In sub-block *B*, we send no fresh information, so each cloud center has only one satellite.

We now proceed to a more formal exposition. For this, we will need some notation. Given a joint distribution $P_{XYZ}$, we denote by $T_{XY}$ the set of all jointly typical sequences (x,y), where the length *n* is understood from the context, and we adopt the δ-convention of [8]. Similarly, given a sequence *z*, $T_{XYZ}\left(z\right)$ stands for the set of all pairs (x,y) that are jointly typical with the given sequence *z*.

To describe the first and last sub-blocks, we define $m_{0}=1$ and $m_{B}=1$, respectively. The proof of the direct part is based on random coding and joint typicality decoding.

#### 4.1.1. Code Construction

We construct *B* codebooks $\left\{C_{b}\right\}$, b∈[1:B], each of length *n*. Each codebook $C_{b}$ is generated randomly and independently of the other codebooks as follows:

- For every b∈[1:B], generate $2^{nR}$ length-*n* cloud centers $\left\{v^{\left(b\right)}\left(j\right)\right\}$, j∈M independently, each with IID $∼P_{V}$ components.
- For every b∈[1:B] and j∈M, generate $2^{nR}$ length-*n* satellites $\left\{u^{\left(b\right)}\left(m|j\right)\right\}$, m∈M conditionally independently given $v^{\left(b\right)}\left(j\right)$, each according to
  (69) $$\prod_{i=1}^{n}P_{U|V}\left(\cdot |v_{i}^{\left(b\right)}\left(j\right)\right).$$

The codebook $C_{b}$ is the collection

(70) $$\left\{v^{\left(b\right)}\left(j\right),u^{\left(b\right)}\left(m|j\right)\right\},\;\left(j,m\right)\in M\times M.$$

Reveal the codebooks to the encoder, decoder, and helper.

#### 4.1.2. Operation of the code

We first describe the operation of the helper and encoder in the first sub-block.

Helper. In the first sub-block, b=1, the helper produces

(71) $$t^{\left(1\right)}=\left(t_{1}^{\left(1\right)},t_{2}^{\left(1\right)},\ldots ,t_{n}^{\left(1\right)}\right)$$

where

(72) $$t_{i}^{\left(1\right)}=h\left(s_{i}^{\left(1\right)},v_{i}^{\left(1\right)}\left(m_{0}\right)\right),\;1\leq i\leq n.$$

Note that $t^{\left(1\right)}$ is causal in $s^{\left(1\right)}$.

Encoder. Set $u^{\left(1\right)}=u^{\left(1\right)}\left(m_{1}|m_{0}\right)$ and $v^{\left(1\right)}=v^{\left(1\right)}\left(m_{0}\right)$. The input to the channel is

(73) $$x^{\left(1\right)}=\left(x_{1}^{\left(1\right)},x_{2}^{\left(1\right)},\ldots ,x_{n}^{\left(1\right)}\right)$$

where

(74) $$\begin{array}{cc} x_{i}^{\left(1\right)} & =f\left(u_{i}^{\left(1\right)}\left(m_{1}|m_{0}\right),v_{i}^{\left(1\right)}\left(m_{0}\right),t_{i}^{\left(1\right)}\left(s_{i}^{\left(1\right)},v_{i}^{\left(1\right)}\left(m_{0}\right)\right)\right)\\ & =f\left(u_{i}^{\left(1\right)},v_{i}^{\left(1\right)},t_{i}^{\left(1\right)}\right),\;1\leq i\leq n. \end{array}$$

Note that $x^{\left(1\right)}$ is causal in $t^{\left(1\right)}$.

Helper at the end of the sub-block. Thanks to its cribbing, at the end of sub-block 1, the helper is cognizant of $x^{\left(1\right)}$. In addition, it knows $v^{\left(1\right)}$ (since it is determined by $m_{0}$, which was set a priori) and $t^{\left(1\right)}$ (since it was produced by itself). The helper now decodes the message $m_{1}$ by looking for an index j∈M such that

(75) $$\left(u^{\left(1\right)}\left(j|m_{0}\right),x^{\left(1\right)}\right)\in T_{UXVT}\left(v^{\left(1\right)},t^{\left(1\right)}\right).$$

If such an index *j* exists and is unique, the helper sets ${\hat{m}}_{1}=j$. Otherwise, an error is declared. By standard results, the probability of error is vanishingly small provided that

(76) R<I(U;X|VT).

Denote by ${\hat{m}}_{1}$ the message decoded by the helper at the end of sub-block 1. We proceed to describe the operation of the helper and encoder in sub-block *b*, when 2≤b≤B−1.

Helper, 2≤b≤B−1. Denote by ${\hat{m}}_{b-1}$ the message decoded by the helper at the end of sub-block (b−1). In sub-block *b*, the helper produces

(77) $$t^{\left(b\right)}=\left(t_{1}^{\left(b\right)},t_{2}^{\left(b\right)},\ldots ,t_{n}^{\left(b\right)}\right)$$

where

(78) $$t_{i}^{\left(b\right)}=h\left(s_{i}^{\left(b\right)},v_{i}^{\left(b\right)}\left({\hat{m}}_{b-1}\right)\right),\;1\leq i\leq n.$$

Encoder, 2≤b≤B−1. Set $u^{\left(b\right)}=u^{\left(b\right)}\left(m_{b}|m_{b-1}\right)$ and $v^{\left(b\right)}=v^{\left(b\right)}\left({\hat{m}}_{b-1}\right)$. The input to the channel is

(79) $$x^{\left(b\right)}=\left(x_{1}^{\left(b\right)},x_{2}^{\left(b\right)},\ldots ,x_{n}^{\left(b\right)}\right)$$

where

(80) $$\begin{array}{cc} x_{i}^{\left(b\right)} & =f\left(u_{i}^{\left(b\right)}\left(m_{b}|m_{b-1}\right),v_{i}^{\left(b\right)}\left(m_{b-1}\right),t_{i}^{\left(b\right)}\left(s_{i}^{\left(b\right)},v_{i}^{\left(b\right)}\left({\hat{m}}_{b-1}\right)\right)\right)\\ & =f\left(u_{i}^{\left(b\right)},v_{i}^{\left(b\right)},t_{i}^{\left(b\right)}\right),\;1\leq i\leq n. \end{array}$$

Note that $t^{\left(b\right)}$ and $x^{\left(b\right)}$ are causal in $s^{\left(b\right)}$ and $t^{\left(b\right)}$, respectively.

Helper at the end of the sub-block, 2≤b≤B−1. At the end of sub-block *b* the helper has $x^{\left(b\right)}$ at hand. In addition, it has $v^{\left(b\right)}\left({\hat{m}}_{b-1}\right)$ (since ${\hat{m}}_{b-1}$ was decoded at the end of the previous sub-block) and $t^{\left(b\right)}$ (since it was produced by itself). The helper now decodes the message $m_{b}$. Assuming that ${\hat{m}}_{b-1}$ was decoded correctly, this can be done with a low probability of error if (37) is satisfied.

We proceed to the last sub-block, where no fresh information is sent. Here $m_{B}=1$, and the operations of the helper and encoder proceed exactly as in (77)–(80), with b=B. Note that in sub-block *B*, the helper need not decode $m_{B}$ since it is set a priori and known to all.

#### 4.1.3. Decoding

At the destination, we employ backward decoding. Starting at sub-block *B* with $m_{B}=1$, the decoder looks for an index j∈M such that

(81) $$\left(u^{\left(B\right)}\left(1|j\right),v^{\left(B\right)}\left(j\right),y^{\left(B\right)}\right)\in T_{UVY}.$$

If such an index exists and is unique, the decoder sets ${\hat{\hat{m}}}_{B-1}=j$. Otherwise, an error is declared. By standard result, the decoding is correct with probability approaching 1 provided

(82) R<I(UV;Y).

In the next (backward) decoding sub-blocks, the decoding proceeds as in (81), with the exception that the estimate ${\hat{\hat{m}}}_{b}$ replaces the default value $m_{B}=1$ in (81). Thus, in sub-block B−1, the decoder has at hand the estimate ${\hat{\hat{m}}}_{B-1}$, and the channel output $y^{\left(B-1\right)}$. It looks for an index *j* such that

(83) $$\left(u^{\left(B-1\right)}\left({\hat{\hat{m}}}_{B-1}|j\right),v^{\left(B-1\right)}\left(j\right),y^{\left(B-1\right)}\right)\in T_{UVY}.$$

Similarly, for 2≤b≤B−1, the decoder looks for an index *j* such that

(84) $$\left(u^{\left(b\right)}\left({\hat{\hat{m}}}_{b}|j\right),v^{\left(b\right)}\left(j\right),y^{\left(b\right)}\right)\in T_{UVY}.$$

If such an index *j* exists and is unique, the decoder sets ${\hat{\hat{m}}}_{b-1}=j$. Otherwise, an error is declared. Assuming that $m_{b}$ was decoded correctly in the previous decoding stage, i.e., ${\hat{\hat{m}}}_{b}=m_{b}$, the decoding of $m_{b-1}$ in sub-block *b* is correct with probability close to 1 provided that (82) holds. Note that $m_{1}$ is decoded in sub-block b=2, that is, $y^{\left(1\right)}$ is not used at the destination. However, the transmission in sub-block 1 is not superfluous, as it is used by the helper to decode $m_{1}$ at the end of the first sub-block. Since (76) and (82) are the two terms in (10), this concludes the proof of the direct part.

### 4.2. Converse Part

Fix |T|, and consider $\left(n,2^{nR},|T|,{\tilde{\epsilon }}_{n}\right)$-codes with ${\tilde{\epsilon }}_{n}↓0$. For each *n*, feed a random message *M* that is drawn equiprobably from the set $\left\{1,2,\ldots ,2^{nR}\right\}$ to the encoder. By the channel model,

(85) $$M⊸\;\text{–}\;\left(X^{n}S^{n}\right)⊸\;\text{–}\;Y^{n}.$$

Fano’s inequality and the fact that ${\tilde{\epsilon }}_{n}↓0$ imply the existence of a sequence $\epsilon_{n}↓0$ for which

(86) $$\begin{array}{ccc} n(R-\epsilon_{n}) & \leq & I(M;Y^{n})\\ & \overset{\left(a\right)}{\leq } & I(M;X^{n}S^{n})\\ & = & I(M;X^{n}|S^{n})\\ & = & \sum_{i=1}^{n}I\left(M;X_{i}|S^{n}X^{i-1}\right)\\ & \overset{\left(b\right)}{=} & \sum_{i=1}^{n}I\left(M;X_{i}|S^{n}X^{i-1}T_{i}\right)\\ & \leq & \sum_{i=1}^{n}I\left(MS_{i}^{n};X_{i}|S^{i-1}X^{i-1}T_{i}\right)\\ & \overset{\left(c\right)}{=} & \sum_{i=1}^{n}I\left(M;X_{i}|S^{i-1}X^{i-1}T_{i}\right)\\ & \leq & \sum_{i=1}^{n}I\left(MY^{i-1};X_{i}|S^{i-1}X^{i-1}T_{i}\right) \end{array}$$

where (a) follows from (85); (b) holds because $T_{i}$ is a function of $X^{i-1}S^{i}$ (8); and (c) holds because $X_{i}$ is a function of $MT^{i}$ and hence of $MS^{i-1}X^{i-1}T_{i}$ (so $I(S_{i}^{n};X_{i}|MS^{i-1}X^{i-1}T_{i})$ must be zero).

We proceed to derive the second bound. Starting again with Fano’s inequality,

(87) $$\begin{array}{ccc} n(R-\epsilon_{n}) & \leq & I(M;Y^{n})\\ & = & \sum_{u=1}^{n}I\left(M;Y_{i}|Y^{i-1}\right)\\ & \leq & \sum_{u=1}^{n}I\left(MY^{i-1};Y_{i}\right). \end{array}$$

Defining

(88) $$\begin{array}{ccc} U_{i} & = & MY^{i-1} \end{array}$$

(89) $$\begin{array}{ccc} V_{i} & = & S^{i-1}X^{i-1} \end{array}$$

we can rewrite (86) and (87) as

(90) $$\begin{array}{ccc} R-\epsilon_{n} & \leq & \sum_{i=1}^{n}I\left(U_{i};X_{i}|V_{i}T_{i}\right) \end{array}$$

(91) $$\begin{array}{ccc} R-\epsilon_{n} & \leq & \sum_{i=1}^{n}I\left(U_{i};Y_{i}\right) \end{array}$$

Moreover, with $U_{i}$ and $V_{i}$ defined as above, $U_{i}V_{i}$ and $S_{i}$ are independent

(92) $$\left(U_{i}V_{i}\right)⊥\;⊥S_{i}$$

and

(93) $$\begin{array}{ccc} T_{i} & = & h_{i}\left(S_{i},V_{i}\right) \end{array}$$

(94) $$\begin{array}{ccc} X_{i} & = & f_{i}\left(U_{i},V_{i},T_{i}\right) \end{array}$$

where $h_{i}$ and $f_{i}$ are (blocklength dependent) deterministic functions. Indeed, $X_{i}$ can be determined from $\left(U_{i},V_{i},T_{i}\right)$ because $U_{i}$ determines the message *M* and $V_{i}$ determined $T^{i-1}$, so $\left(U_{i},V_{i},T_{i}\right)$ determines $\left(M,T^{i}\right)$ from which $X_{i}$ can be computed using (5).

We next do away with the sums by conditioning on a time-sharing random variable. Let *Q* be a random variable uniformly distributed over {1,2,…,n} independently of the channel and the state. Using *Q*, we can express the bounds (90), (91) as

(95) $$\begin{array}{ccc} R-\epsilon_{n} & \leq & I(U_{Q};X_{Q}|V_{Q}T_{Q}Q)\\ & = & I(U_{Q}Q;X_{Q}|V_{Q}T_{Q}Q)\\ & = & I(\tilde{U};X|VT)\\ & = & I(\tilde{U}V;X|VT)\\ & = & I(U;X|VT) \end{array}$$

(96) $$\begin{array}{ccc} R-\epsilon_{n} & \leq & I(U_{Q};Y_{Q}|Q)\\ & \leq & I(U_{Q}Q;Y_{Q})\\ & = & I(\tilde{U};Y)\\ & \leq & I(\tilde{U}V;Y)\\ & = & I(U;Y) \end{array}$$

where we define

(97) $$X=X_{Q},\;Y=Y_{Q},\;T=T_{Q},\;S=S_{Q}$$

and the auxiliaries

(98) $$\begin{array}{ccc} V & = & \left(V_{Q}Q\right) \end{array}$$

(99) $$\begin{array}{ccc} \tilde{U} & = & \left(U_{Q}Q\right) \end{array}$$

(100) $$\begin{array}{ccc} U & = & \left(\tilde{U},V\right)=\left(U_{Q}V_{Q}Q\right). \end{array}$$

Note that the conditional law of *Y* given (XTS) is that of the channel, namely, $W_{Y|XS}$ and that *S* is distributed like the channel state. Moreover,

(101) V⊸– U⊸– (XTS)⊸– Y.

Since *U* and *V* contain the time sharing random variable *Q*, (93) and (94) imply that,

(102) $$\begin{array}{ccc} T & = & h(S,V) \end{array}$$

(103) $$\begin{array}{ccc} X & = & \tilde{f}\left(\tilde{U},V,T\right)=f\left(U,T\right) \end{array}$$

for some deterministic functions *h* and *f*. Therefore, the joint distribution under which the RHS of (95) and the RHS of (96) are computed is of the form

(104) $$P_{S\tilde{U}VTXY}=P_{S}\;P_{\tilde{U}V}\;P_{T|SV}P_{X|\tilde{U}VT}\;W_{Y|XS}$$

where $P_{T|SV}$ and $P_{X|\tilde{U}VT}$ are zero-one laws, or

(105) $$P_{SUVTXY}=P_{S}\;P_{U}\;P_{V|U}\;P_{T|SV}\;P_{X|UT}\;W_{Y|XS}$$

where $P_{T|SV}$, $P_{X|UT}$, and $P_{V|U}$ are zero-one laws.

The form (105) and the inequalities (95), (96) establish the converse.

### 4.3. Cardinality Bounds

We next proceed to bound the alphabet sizes of U,V in two steps. In the first, we do so while relaxing the zero-one-law requirements. In the second, we enlarge the alphabet to fulfill said requirements. Let

(106) L=|X||T||S|+1.

Fix a conditional distribution p(x,t,s|u), and define the *L* functions of p(u|v):

(107) $$\begin{array}{ccc} p(x,t,s|v) & = & \sum_{u}p\left(x,t,s|u\right)\;p\left(u|v\right)\;\;(L-2\mathrm{functions}) \end{array}$$

$$\begin{array}{c} I(U;X|T,V=v) \end{array}$$

$$\begin{array}{c} I(U;Y|V=v) \end{array}$$

(with the L−2 functions corresponding to all by one of the tuples (x,t,s)). By the support lemma [5,8], there exists a random variable $V^{\prime }$ with alphabet $|V^{\prime }|\leq L$, such that $P_{XTS}$, I(U;X|TV), and I(U;Y) are preserved. Denote by $U^{\prime }$ the resulting random variable *U*, i.e.,

(108) $$P_{U^{\prime }}\left(u^{\prime }\right)=\sum_{v^{\prime }}p\left(u^{\prime }|v\right)P_{V^{\prime }}\left(v^{\prime }\right).$$

We next bound the alphabet size of $U^{\prime }$. For each $v^{\prime }\in V^{\prime }$, we define the *L* functions

(109) $$\begin{array}{c} p(x,t,s|v^{\prime },u^{\prime })\;(L-2\mathrm{functions}) \end{array}$$

(110) $$\begin{array}{c} I(U^{\prime };X|T,V^{\prime }) \end{array}$$

(111) $$\begin{array}{c} I(U^{\prime };Y|V^{\prime }). \end{array}$$

Applying again the support lemma, for every $v^{\prime }$ there exists a random variable $U^{\prime \prime }$ with alphabet $|U^{\prime \prime }|\leq L$ such that (109)–(111) are preserved. If we multiply $U^{\prime \prime }$$|V^{\prime }|$ times we can, with proper labeling of the elements of $U^{\prime \prime }$, retain a Markov structure like (101). Now the alphabet sizes are fixed and independent of *n*. Thus, substituting $V^{\prime },U^{\prime \prime }$ in (95), (96) and taking the limit n→∞ we have the upper bound

(112) $$\begin{array}{ccc} R & \leq & I(U^{\prime \prime };X|V^{\prime }T) \end{array}$$

(113) $$\begin{array}{ccc} R & \leq & I(U^{\prime \prime };Y) \end{array}$$

where

(114) $$\begin{array}{ccc} P_{SU^{\prime \prime }V^{\prime }TXY} & = & P_{S}\;P_{U^{\prime \prime }V^{\prime }}\;P_{T|SV^{\prime }}\;P_{X|U^{\prime \prime }V^{\prime }T}\;W_{Y|XS} \end{array}$$

(115) $$\begin{array}{c} |V^{\prime }\left|\leq L,\;\right|U^{\prime \prime }|\leq L^{2} \end{array}$$

and the following Markov chain holds:

(116) $$V^{\prime }⊸\;\text{–}\;U^{\prime \prime }⊸\;\text{–}\;\left(XTS\right)⊸\;\text{–}\;Y.$$

Note, however, that $P_{T|SV^{\prime }}$ and $P_{X|U^{\prime \prime }V^{\prime }T}$ are no longer zero-one laws. We remedy this using the Functional Representation lemma (FRL) [5] (at the cost of increasing the alphabet sizes): a standard convexity argument will not do because—although I(U;X|VT) is a convex function of $P_{T|SV}$ and also a convex function of $P_{X|UVT}$ and likewise I(U;Y)—the minimum of two convex functions need not be convex.

The Functional Representation lemma implies that—without altering the conditional law of *T* given $SV^{\prime }$ nor of *X* given $U^{\prime \prime }V^{\prime }T$—the random variables *T* and *X* can be represented as

(117) $$\begin{array}{ccc} T & = & {\tilde{g}}_{1}\left(SV^{\prime },Z_{1}\right) \end{array}$$

(118) $$\begin{array}{ccc} X & = & {\tilde{g}}_{2}\left(U^{\prime \prime }V^{\prime }T,Z_{2}\right) \end{array}$$

where ${\tilde{g}}_{1}$, ${\tilde{g}}_{2}$ are deterministic functions; $Z_{1}$ and $Z_{2}$ are independent random variables that are independent of $\left(SV^{\prime },U^{\prime \prime }V^{\prime }T\right)$; and their alphabets satisfy

(119) $$\begin{array}{ccc} |Z_{1}| & \leq & \left|S|\;\right|V^{\prime }|\;\left(|T|-1\right)+1 \end{array}$$

(120) $$\begin{array}{ccc} |Z_{2}| & \leq & |U^{\prime \prime }\left|\;\right|V^{\prime }|\;|T|\;\left(|X|-1\right)+1. \end{array}$$

At the expense of increased alphabets sizes, we now append $Z_{1}$ to $V^{\prime }$ and $Z_{2}$ to $U^{\prime \prime }$ to form the new auxiliary random variables

(121) $$\begin{array}{ccc} \hat{V} & = & \left(V^{\prime }Z_{1}\right) \end{array}$$

(122) $$\begin{array}{ccc} \hat{U} & = & \left(U^{\prime \prime }Z_{2}\right) \end{array}$$

with alphabet sizes

(123) $$\begin{array}{ccc} |\hat{V}| & \leq & \left|S|\right|V^{\prime }{|}^{2}\left(|T|-1\right)+\left|V^{\prime }\right| \end{array}$$

(124) $$\begin{array}{ccc} |\hat{U}| & \leq & |U^{\prime \prime }{|}^{2}|V^{\prime }\left||T\right|\left(|X|-1\right)+\left|U^{\prime \prime }\right|. \end{array}$$

We set

(125) $$P_{X|\hat{U}\hat{V}T}\left(x|u^{\prime \prime },z_{2},v^{\prime },z_{1},t\right)=1\left\{x={\tilde{g}}_{2}\left(u^{\prime \prime },z_{2},v^{\prime },t\right)\right\}$$

(irrespective of $z_{1}$) and

(126) $$P_{T|\hat{V}S}\left(t|v^{\prime },z_{1},t\right)=1\left\{t=g_{1}\left(s,v^{\prime },z_{1}\right)\right\}$$

where 1{statement} equals 1 if the statement is true and equals 0 otherwise.

As we next argue, these auxiliary random variables and the above zero–one laws do not decrease the relevant mutual information expressions.

Beginning with $I(\hat{U};X|\hat{V}T)$, we note that $H\left(X|\hat{V}T\right)=H\left(X|V^{\prime }T\right)$ because we have preserved the joint law of $V^{\prime }T$ and because $Z_{1}$ does not influence the mapping (54) to *X*. Since $H\left(X|U^{\prime \prime }Z_{2}VT\right)\leq H\left(X\right|H\left(X|U^{\prime \prime }VT\right)$, this establishes that

(127) $$I\left(\hat{U};X|\hat{V}T\right)\geq I\left(U^{\prime \prime };X|V^{\prime }T\right).$$

Likewise, our new auxiliary random variables and zero–one laws do not alter H(Y), but $H\left(Y|\hat{U}\right)\leq H\left(Y|U^{\prime \prime }\right)$, so

(128) $$I\left(\hat{U};Y\right)\geq I\left(U^{\prime \prime };Y\right).$$

This completes the proof of Theorem 1.

## Data Availability

No new data were created or analyzed in this study. Data sharing is not applicable to this article.

## Abbreviations

The following abbreviations are used in this manuscript:

IID	Independent and Identically Distributed
FRL	Functional Representation Lemma
PMF	Probability Mass Function
RHS	Right-Hand Side
